# 

**Published:** 1888-03

**Authors:** 


### The Chicago Medical Journal and Examiner.

Volume 56. Number 3.

### CONTENTS OF THIS NUMBER.

**Original Communications—** PAGE.

Gunshot Wounds of the Abdomen. J. B. Murphy ... 129
Ophthalmia Neonatorum. R. Tilley ... 134
Poisoning by Ingestion of One Grain of Atropia Sulphate. C. D. Wescott ... 138

**Editorial—**

National Control of Maritime Quarantine ... 139
The American Medical Association ... 141
Fraudulent Medical Adverti.ing ... 142
Medical Progress in Italy ... 142

**Society Reports—**

Gynæcological Society of Chicago ... 143
Baltimore Gynæcological and Obstetrical Society ... 151

**Notes from Foreign Medical Societies—**

Pathological Society of London ... 160
Obsterical Society of London ... 162
Harveian Society of London ... 164
Royal Medical and Chirurgical Society of London ... 164
Medical Society of London ... 166
Medico-Chirurgical Society of Edinburgh ... 168
Royal Academy of Medicine in Ireland ... 169
South Indian Branch of the British Medical Association ... 170
Clinical Society of Manchester ... 170

**Foreign Correspondence—**

Genoa ... 171

**Extracts and Abstracts—**

Michigan State Board of Health ... 177
The Medical Corps of the United States Navy ... 180
Quarantine Does not Deal with the Most Dangerous Diseases ... 181
Fogs and their Effects ... 182
An Extraordinary Injury ... 182
Guy's Hospital Reports ... 183
Ano-Vesicle Centre ... 192
Brain Surgery ... 193
Uræmic Convulsions treated by Pilocarpine ... 194
The Action of Naphthaline on the Visual Organs ... 195

**Book Reviews** ... 196

**Miscellaneous—**

Import Duties on Surgical Instruments ... 198
The William F. Jenks Memorial Prize ... 198
Lanolin and Its Uses ... 199
Rush Medical College Commencement ... 199
Proposed Laryngological Society ... 199
Unmerited Syphilis ... 200
The Disciplinary Powers of the Royal College of Physicians ... 200
The American Anthropologist ... 200

**Announcements—**

The American Medical Association ... 200
The Illinois State Medical Society ... 200
The German Society of Naturalists and Physicians ... 200
International Exhibition of Hygiene ... 200
The Fourth International Otological Congress ... 200

